# Quantification of attenuation and speckle features from endoscopic OCT images for the diagnosis of human brain glioma

**DOI:** 10.1038/s41598-024-61292-z

**Published:** 2024-05-10

**Authors:** P. V. Aleksandrova, K. I. Zaytsev, P. V. Nikitin, A. I. Alekseeva, V. Y. Zaitsev, K. B. Dolganov, I. V. Reshetov, P. A. Karalkin, V. N. Kurlov, V. V. Tuchin, I. N. Dolganova

**Affiliations:** 1 Prokhorov General Physics Institute of the Russian Academy of Sciences, Moscow, Russia 119991; 2 Department of Biomedical Engineering, University of Houston, Houston, TX USA; 3 N.N. Burdenko National Medical Research Center for Neurosurgery, Moscow, Russia 125047; 4 Avtsyn Research Institute of Human Morphology, FSBSI “Petrovsky National Research Centre of Surgery”, Moscow, Russia 117418; 5 A.V. Gaponov-Grekhov Institute of Applied Physics of the Russian Academy of Sciences, Nizhny Novgorod, Russia 603950; 6 Institute for Cluster Oncology, Sechenov First Moscow State Medical University, Moscow, Russia 119991; 7 Osipyan Institute of Solid State Physics of the Russian Academy of Sciences, Chernogolovka, Russia 142432; 8 Science Medical Center, Saratov State University, Saratov, Russia 410000; 9 Institute of Precision Mechanics and Control, FRC “Saratov Scientific Centre of the Russian Academy of Sciences”, Saratov, Russia 410028; 10 Tomsk State University, Tomsk, Russia 634050

**Keywords:** Biophotonics, Surgical oncology, Imaging and sensing

## Abstract

Application of optical coherence tomography (OCT) in neurosurgery mostly includes the discrimination between intact and malignant tissues aimed at the detection of brain tumor margins. For particular tissue types, the existing approaches demonstrate low performance, which stimulates the further research for their improvement. The analysis of speckle patterns of brain OCT images is proposed to be taken into account for the discrimination between human brain glioma tissue and intact cortex and white matter. The speckle properties provide additional information of tissue structure, which could help to increase the efficiency of tissue differentiation. The wavelet analysis of OCT speckle patterns was applied to extract the power of local brightness fluctuations in speckle and its standard deviation. The speckle properties are analysed together with attenuation ones using a set of ex vivo brain tissue samples, including glioma of different grades. Various combinations of these features are considered to perform linear discriminant analysis for tissue differentiation. The results reveal that it is reasonable to include the local brightness fluctuations at first two wavelet decomposition levels in the analysis of OCT brain images aimed at neurosurgical diagnosis.

## Introduction

Optical coherence tomography (OCT) is recognized as a fast, noninvasive and label-free method for imaging of biological tissues, providing information about their internal structure and properties^1–5^. Therefore, OCT has found its application in such areas as ophthalmology, oncology, dermatology, and vascular surgery^6–9^. Recently, OCT has shown a high potential in experimental medicine and various clinical fields, including neurosurgery^10–13^.

Beginning with the demonstration of the ability to visualize melanoma metastasis^14^, the further studies revealed a huge potential of OCT to become a highly informative diagnostic technique in surgery of glial^15–24^ and meningioma brain tumors^25^. It was shown that OCT allows distinguishing between normal brain tissue and tumor and detecting of the benign or malignant nature of the tumor. Kut et al.^21^ developed an OCT-based method of optical attenuation analysis for human brain tissues. This work demonstrated the differences between optical scattering properties of intact and tumorous tissues as well as between those of gray and white brain matter. Bohringer et al.^19^ showed the differences of attenuation properties extracted from OCT images of normal brain, areas of necrosis, solid tumor and tumor infiltration. The research of Yashin et al.^22^ involving 176 ex vivo human specimens from 30 patients with brain glioma and in vivo studies from 17 patients revealed the advantage of cross-polarization OCT (CP-OCT) for differentiating tumor and non-tumor tissues using signal intensity. At the same time, OCT was successfully applied for imaging of the boundaries of epileptic focus on the experimental mice model^26^. Meanwhile, a number of research involved animal models of brain tumor, such as orthotopic mouse model G112^18^ and rat model 101.8^24,27,28^, for study OCT potential for neurosurgery.

Most existing methods for OCT signal processing aimed at neurosurgical diagnosis are based on the analysis of signal intensity and extraction of the attenuation or scattering coefficients. The obtained experimental data indicate a decrease in the attenuation coefficient for glioma tissue compared to intact brain tissue, which is usually considered to be white matter and cortex^21,23,24^. However, the presence of necrosis in such tissues as glioblastoma increases light scattering and the attenuation coefficient^23,24^. Due to the morphological characteristics of white matter, namely, the presence of nerve fibers, which are covered with the myelin sheath, the attenuation coefficient is characterized by high values^29^. On the contrary, cortex yields less intense and slowly attenuating OCT signal, which makes this type of tissue similar to tumors^30^. Thus, considering the label-free nature of OCT along with the huge diversity of measurement conditions and tissue properties, there is a need for further development of OCT image processing algorithms, which would help to increase their current sensitivity and specificity.

One of the possible ways to improve the performance of OCT-based neurodiagnosis is the account of the speckle structures, appeared in OCT images due to the interference of a large number of elementary waves with random phases arising from spatially coherent light propagation through a turbid medium. Since most biological tissues are heterogeneous, when illuminated with coherent light, there will always be a speckle structure that can both distort measurements and at the same time provides new information about the tissue structural properties^31–33^.

Previously, the speckle-based analysis of OCT images has been applied for characterization of tissue properties, such as the dynamics of moving scatterers in a sample^34–36^. Besides, it was used for tissue classification. Mcheik et al.^37^ proposed a comparative analysis of speckle structures in OCT images including several probability density functions (PDFs) to distinguish skin layers; the Nakagami distribution showed the best results. De Jesus et al.^38^ applied the same method to distinguish the groups of corneal data and demonstrated the potential of the Generalized Gamma distribution for the speckle statistics. Thus, assuming that speckle patterns are associated with tissue properties, their analysis could be included in the existing algorithms for OCT-based differentiation of brain tissues. Thereby, the main scope of our work is to investigate, whether speckle properties can provide benefit for brain glioma and intact tissues differentiation.

For this aim, we consider the OCT images of ex vivo intact and malignant human brain tissue specimens, obtained by the endoscopic OCT system^24^. Data set includes human gliomas of the World Health Organization (WHO) 1–4 grades and intact cortex and white matter. We propose an extraction of the speckle-based features, i.e. the power of local brightness fluctuations in speckle patterns and its standard deviation, from OCT images using wavelet analysis (WA) and study their applicability for distinguishing between glioma and intact brain tissues. Finally, we perform the tissue distinguishing by means of linear discriminant analysis (LDA) and different combinations of features, which are related to attenuation and speckle properties, assuming the maximization of sensitivity ($\textrm{Se}$) and specificity ($\textrm{Sp}$). We compare $\textrm{Se}$, $\textrm{Sp}$ and precision ($\textrm{Pr}$) estimated for the considered combinations of features. The results of this study revealed the advantages and drawbacks of these combinations for OCT-aided brain glioma diagnosis.

## Results

### OCT of tissue samples

Gliomas are intracranial tumors that usually affect the functioning parts of the brain and cause higher mortality and morbidity rate than other forms of tumors of the central nervous system (CNS)^39,40^. It is one of the most common CNS tumor type. According to the 2021 revision, WHO classification of CNS tumors includes four nosologies^41^:

- Grade 1 – benign tumor: pilocytic astrocytoma, subependymal giant cell astrocytoma;
- Grade 2 – highly differentiated gliomas including astrocytoma with a mutation in the isocitrate dehydrogenase 1 (IDH1) or 2 (IDH2) genes, oligodendroglioma with a mutation and codeletion 1p/19q;
- Grade 3 – diffuse astrocytoma and oligodendroglioma with mutation in the IDH1 or IDH2 genes;
- Grade 4 – glioblastoma without mutation in the IDH1 or IDH2 genes, astrocytoma with mutation.

**Table 1:** Human brain tissue samples.

$\#$	Tissue type	Number of patients	Gender/age	WHO grade	Number of B-scans
1	Perifocal cortex	2	M / 66	−	12
			F / 69		
2	Perifocal white matter	2	M / 45	−	15
			M / 38		
3	Pilocytic astrocytoma	3	F / 39	1	22
			F / 31		
			F / 25		
4	Diffuse astrocytoma	5	M / 29	2	44
			F / 63		
			M / 31		
			M / 43		
			F / 56		
5	Oligodendroglioma	3	F / 47	3	17
			F / 26		
			M / 41		
6	Glioblastoma	6	F / 69	4	103
			M / 52		
			F / 64		
			F / 69		
			F / 55		
			F / 20		

**Figure 1:**
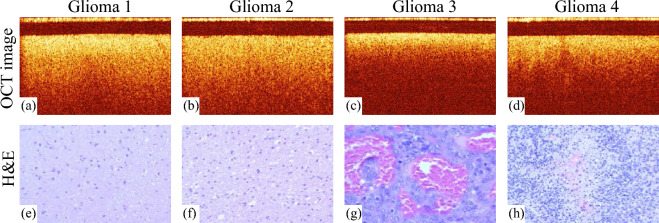
Representative OCT images of the ex vivo human glioma tissue of 1–4 grades (**a**–**d**) and H&E histological images of the same tissue types (**e**–**h**).

In our study, we considered ex vivo samples of all glioma grades and perifocal brain tissue. For details see Table 1. The samples were excised during neurosurgery, according to the initial medical diagnosis. OCT imaging was performed no later than 4 hours after surgery. For this aim, we used the time-domain common-path endoscopic OCT system OCT1300Y designed at IAP RAS and described in our previous work^24^. It operates at 1.3 $\mu$m central wavelength and is equipped with a flexible endoscopic probe. The average output power is 0.6 mW. OCT images have 400$\times$256 pixels, that corresponds to the approximately 1.96-mm-width and 1-mm-depth tissue sample scanned with the resolution 20 $\mu$m in lateral direction and 24 $\mu$m in axial direction (in air). To protect the samples from dehydration during transportation and imaging, they were covered with gelatin films. After OCT imaging, the samples were fixed with formalin for the further Hematoxylin and Eosin (H &E)-stained histology that confirmed the initial diagnosis. The representative OCT and histological images of glioma tissues are shown in Fig. 1.

The samples of freshly excised tumor tissue and histological preparations for research were obtained from the N.N. Burdenko National Medical Research Center for Neurosurgery after obtaining informed consents from patients and approval of the study protocol by the ethics committee of the N.N. Burdenko National Medical Research Center for Neurosurgery. The samples were excised during neurosurgery, performed in accordance with the initial medical diagnosis. All experiments with thus excised ex vivo tissue samples were performed in accordance with the relevant guidelines and regulations.

**Figure 2:**
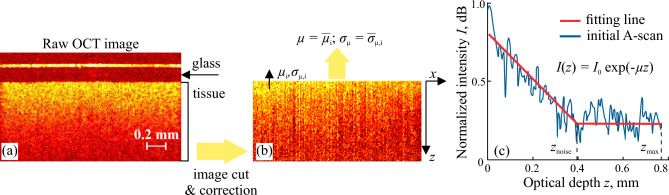
OCT-images of the ex vivo human brain tissue; (**a**) a representative example of “raw” OCT image of glioma grade 1; (**b**) final image of ROI after cut and correction of the “raw” image; (**c**) extraction of $\mu$ from the normalized intensity of the A-scan *I*(*z*).

Prior to perform the analysis of OCT images and extract tissue optical and speckle features, we applied a pre-processing procedure. At the first stage, we perform the calibration of our system using intralipid aqueous solution as described in Ref.^24^ It helped us to experimentally account the point spread function *h*(*z*) of the used OCT system, which has the influence on the measured reflected intensity as $I_{\textrm{m}}(z) = I_0\exp {(-\mu z)} \cdot h(z)$, and adjust the measurement of tissue attenuation. After that the intensity was normalized using the averaged signal from the interface between the OCT probe and cover glass, on which the sample was fixed. Then we get and aligned the region of interest (ROI), which corresponded to the tissue layer (see Fig. 2a). The upper layers in OCT images, originated from the OCT probe and the glass, were cut off. Alignment by the tissue–glass interface helped to correct the possible uneven gap between the probe and the glass. Then we corrected the image distortion by removing the side 15 pixels. After thus made correction, the depth distribution of *I*(*z*) differs from the measured one, as it can be noticed from Fig. 2b.

As it is clear from Table 1, within each set of OCT images referred to a certain tissue type, several B-scans correspond to the same patient. To eliminate the possible impact of inter-correlation between such images on the overall result of tissue differentiation, we checked the cross-correlation between all images from a certain set. The obtained values of cross-correlation coefficient for images received from each patient [0.55..0.86] do not exceed 0.9, which is the maximal value of this coefficient for images received from different patients. At the same time, the average coefficients for such combinations were comparable, both 0.77. Therefore, the result of thus made verification allowed us to use entire set of images for further extraction of features and perform tissue differentiation.

### Analysis of attenuation properties

From the prepared images, we estimated the attenuation coefficient $\mu _i$ of ith A-scan, that is the sum of scattering $\mu _{\textrm{s}}$ and absorption $\mu _{\textrm{a}}$ coefficients. Since in many biological tissues light scattering mostly determines the signal attenuation^42^, it is often considered that $\mu \simeq \mu _{\textrm{s}}$.

In the single scattering approximation^24,43–45^, the intensity of OCT signal exponentially decays with the sample optical depth *z*

1 $$\begin{aligned} I(z) = I_{0}\exp (-\mu _i z), \end{aligned}$$

where $I_{0}$ is the maximum peak of the signal intensity received from the tissue upper interface for ith A-scan. To extract $\mu _i$ as a slope of the A-scan (2c), we exclude the part of the signal from $z_{\textrm{noise}}$ to $z_{\textrm{max}}$ that corresponds to the noise level, determining $z_{\textrm{noise}}$ by the minimization of mean square error of fitting

2 $$\begin{aligned} z_{\textrm{noise}} = {\textrm{argmin}}_{\textrm{z}} \Bigg [ \frac{\sum _{0}^{z_{\textrm{noise}}} [I(z)-I_{\textrm{fit}}(z,\mu _i)]^2}{N_{\textrm{decay}}} +\frac{\sum _{z=z_{\textrm{noise}}}^{z_{\textrm{max}}} [I(z)-I_{\textrm{noise}}]^2}{N_{\textrm{noise}}}\Bigg ] \end{aligned}$$

where $I_{\textrm{fit}}(z,\mu _i)$ is the sloped fit line, $I_{\textrm{noise}} = I_{\textrm{fit}}(z_{\textrm{noise}})$ is the horizontal fit line corresponding to noise level, $N_{\textrm{decay}}$ and $N_{\textrm{noise}}$ are the numbers of terms in two corresponding regions of the A-scan.

**Figure 3:**
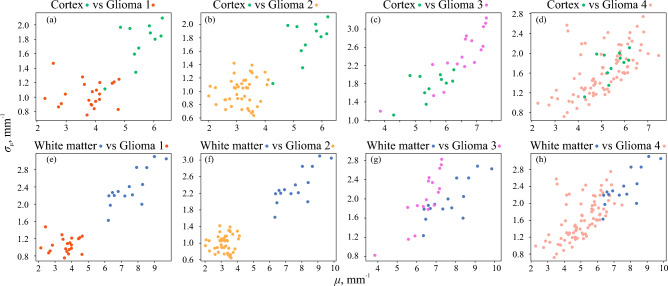
Distribution of the attenuation properties $\mu$ and $\sigma _{\mathrm {\mu }}$ of the analyzed set of ex vivo brain tissue samples. The dots are colored in accordance with the H&E-stained histology of the corresponding tissue samples.

In addition to the attenuation coefficient, its diversity within the sample also yields the information about optical properties of the tissue, describing their heterogeneity originated due to the possible vascularization or the presence of hemorrhages, necrosis, and cysts in glioma tissue. Thus, we estimated the standard deviation of $\mu _i$ within the small region in the lateral direction, which includes several neighboring A-scans $N_{\textrm{A}}$

3 $$\begin{aligned} \sigma _{\mu ,i} = \left[ \sum _{i=1}^{N_{\textrm{A}}} (\mu _i - {\overline{\mu }})^2/N_{\textrm{A}} \right] ^{0.5}, \end{aligned}$$

where ${\overline{\mu }}$ is the mean value of $\mu _i$ in the region of analysis, which was chosen to be equal to $150~\mu$m, so that $N_{\textrm{A}} = 30$, that is comparable with the typical size of vessel in a brain tissue. Finally, $\mu _i$ and $\sigma _{\mu ,i}$ were averaged within each image (Fig. 2b) to get $\mu$ and $\sigma _{\mu }$. These parameters form the space of optical properties, which we use in the analysis of difference between glioma and intact tissue.

The distribution of $\mu$ and $\sigma _{\mathrm {\mu }}$ for our set of samples is shown in Fig. 3. The dots are colored in accordance with the H&E-stained histology of the corresponding tissue samples. Despite the limited number of patients and tissue samples, we can notice significant overlap between the dot clouds in some pairs of tissue types. It is clear that discrimination between intact brain tissue and high-grade glioma is problematic. This fact indirectly confirms the need for additional features, that can improve the OCT-based glioma diagnosis.

### Wavelet-based analysis of OCT speckle pattern

WA is widely used for enhancement and de-noising of OCT images^46^, as well as for their background subtraction and object recognition^47^. Its effectiveness is due to the fact that the basic functions of the wavelet transformation have properties similar to those of wave packets: zero mean value, limitation and high localization in both time and frequency domains. The feasibility of using WA in OCT stems from the ability to effectively suppress noise in multi-scale images, as well as the ability to suppress speckle noise and smooth the image without significant blurring the details^48^.

Essock et al.^49^ studied the possibility of using WA of OCT images to identify glaucoma. Lingley-Papadopoulos et al.^50^ demonstrated for the first time the ability of applying a combination of wavelet and texture analysis of OCT images to detect cancer tissue. WA often helps to reduce speckle noise background and to enhance obscured patterns, improving signal to noise ratio^51^. At the same time, applying wavelet-based image processing, the resolvable features of OCT image can be effectively separated from speckle pattern^52^. Thus, WA is an effective instrument for extraction and characterization of speckle patterns from OCT images.

In this work, we applied discrete wavelet transformation, which is based on the decomposition of a signal into a weighted linear combination of wavelet functions and scaling functions. Both functions are orthogonal and divide the function space into high and low frequency spaces. Each level of decomposition yields two sets of coefficients, namely, a detail coefficient set and an approximation coefficient set. The detail coefficients, which are associated with wavelet function, capture high-frequency information. The approximation coefficients, which are associated with the scaling function, capture with low-frequency information. Since in this work it is necessary to analyse speckle distribution in OCT images and their ability to carry out valuable data, only detail coefficients are considered.

**Figure 4:**
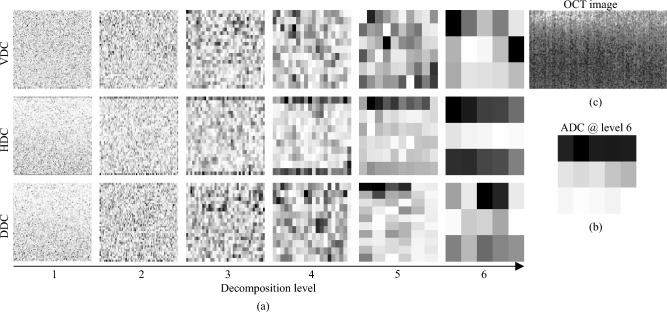
An example of B-scan wavelet decomposition. (**a**) Detail coefficients DDC, HDC, VDC at different decomposition levels from 1 to 6; (**b**) approximation coefficients at the 6th level; (**c**) decomposition is shown for the corrected OCT image of glioma grade 4.

In our study, we applied the wavelet filter *bior3.5* from the biorthogonal family to analyze OCT images of brain tissue. The biorthogonal basis function is used to avoid a possible distortion at the edge^53^. The efficiency of the particular filter *bior3.5* for the applied OCT system was demonstrated previously^54^. However, it should be mentioned that the choice of the wavelet filter mainly depends on the OCT system type. Thus, another type of system may need to switch to another wavelet filter.

It was shown that the 1st and 2nd decomposition levels mostly yield the information about the speckle pattern in OCT images^55^, while higher levels reflect the noise data. Thus, in our work, the detail coefficients were collected for each OCT image at first two decomposition levels. Figure 4 illustrates the transformation of vertical (VDC), horizontal (HDC) and diagonal (DDC) detail coefficients at first six levels for a representative OCT image of glioma grade 4.

Next, the power of local brightness fluctuations in the detail coefficients was introduced as a first parameter connected with a speckle pattern

4 $$\begin{aligned} P_{\textrm{a}} = \frac{1}{K} \Bigg [ \sum \limits _{n_{\textrm{X}},n_{\textrm{Y}}} |I^{\textrm{HDC}}_{\textrm{a}} (n_{\textrm{X}},n_{\textrm{Y}})|^2 + \sum \limits _{n_{\textrm{X}},n_{\textrm{Y}}} |I^{\textrm{VDC}}_{\textrm{a}} (n_{\textrm{X}},n_{\textrm{Y}})|^2 + \sum \limits _{n_{\textrm{X}},n_{\textrm{Y}}} |I^{\textrm{DDC}}_{\textrm{a}} (n_{\textrm{X}},n_{\textrm{Y}})|^2 \Bigg ], \end{aligned}$$

where $K = N_{\textrm{X}} \times N_{\textrm{Y}}$ is a factor which takes into account the number of pixels in two coordinates; $n_{\textrm{X}}$ and $n_{\textrm{Y}}$ are current pixel number; $I^{\textrm{HDC}}_{\textrm{a}}$, $I^{\textrm{VDC}}_{\textrm{a}}$, $I^{\textrm{DDC}}_{\textrm{a}}$ are the intensities of horizontal, vertical and diagonal detail coefficients after image decomposition at the level $a=[1,2]$, respectively.

In addition to the power of local brightness fluctuations in speckle, the standard deviation of $P_{\textrm{a}}$ within the final size of decomposed image $N_{\textrm{X}}^*$, $N_{\textrm{Y}}^*$ in the lateral direction was obtained

5 $$\begin{aligned} \sigma _{\textrm{Pa}} = \left[ \sum \limits _{n_{\textrm{X}}=1}^{N_{\textrm{X}}^*} (P_{\textrm{a}}^{\textrm{X}}(n_{\textrm{X}}) - P_{\textrm{a}})^2 / N_{\textrm{X}}^*\right] ^{0.5} \end{aligned}$$

where $P_{\textrm{a}}^{\textrm{X}}(n_{\textrm{X}})$ is the mean power in $\textrm{Y}$ direction for each pixel number $n_{\textrm{X}}$

6 $$\begin{aligned} P_{\textrm{a}}^{\textrm{X}}(n_{\textrm{X}}) = \frac{1}{N_{\textrm{Y}}} \Bigg [ \sum \limits _{n_{\textrm{Y}}} |I^{\textrm{HDC}}_{\textrm{a}} (n_{\textrm{X}},n_{\textrm{Y}})|^2 + \sum \limits _{n_{\textrm{Y}}} |I^{\textrm{VDC}}_{\textrm{a}} (n_{\textrm{X}},n_{\textrm{Y}})|^2 + \sum \limits _{n_{\textrm{Y}}} |I^{\textrm{DDC}}_{\textrm{a}} (n_{\textrm{X}},n_{\textrm{Y}})|^2 \Bigg ]. \end{aligned}$$

In the described way, similarly to the optical parameters, $P_{\textrm{a}}$ and $\sigma _{\textrm{Pa}}$ form the space of speckle parameters, used further for tissue analysis.

**Figure 5:**
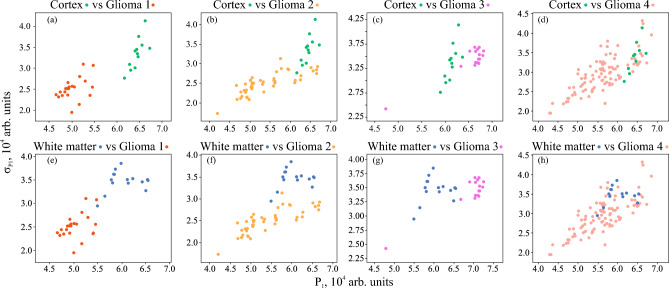
Distribution of the speckle properties $P_{\textrm{1}}$ and $\sigma _{\textrm{P1}}$ of the analyzed set of ex vivo brain tissue samples. The dots are colored in accordance with the H&E-stained histology of the corresponding tissue samples.

**Figure 6:**
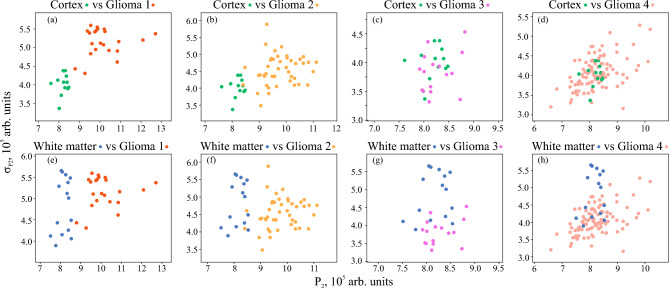
Distribution of the speckle properties $P_{\textrm{2}}$ and $\sigma _{\textrm{P2}}$ of the analyzed set of ex vivo brain tissue samples. The dots are colored in accordance with the H&E-stained histology of the corresponding tissue samples.

The distributions of thus obtained properties using the 1st and 2nd decomposition levels are shown in Figs. 5 and 6. It is obvious that in several pairs of tissue types, the application of the considered speckle features results in rather good localization of dot clouds. Meanwhile, there are also evident drawbacks. For example, one can notice the overlap of dot clouds when glioma grade 4 is considered. Nevertheless, these results make it reasonable to further consider speckle properties as the possible features for brain tissue differentiation. Thus, to combine the advantages of two analyzed approaches, they can be merged by formation of various feature spaces.

### Combinations of attenuation and speckle features for discrimination of glioma tissue

The combinations of features considered in this work are listed in Tables 2 and 3. We compare the performance of single features, namely $\mu$, $P_1$, $P_2$, then consider combinations of them with the corresponding dispersion in 2D feature space $[\mu , \sigma _\mathrm {\mu }]$, $[P_1, \sigma _{\textrm{P1}}]$, $[P_2, \sigma _{\textrm{P2}}]$. Moreover, we combine the attenuation features with the speckle ones $[\mu , P_1]$, $[\mu , P_2]$, $[\mu , \sigma _\mathrm {\mu }, P_1]$, $[\mu , \sigma _\mathrm {\mu }, P_2]$, $[\mu , P_1, P_2]$, $[\mu , \sigma _\mathrm {\mu }, P_1, \sigma _{\textrm{P1}} ]$, $[\mu , \sigma _\mathrm {\mu }, P_2, \sigma _{\textrm{P2}} ]$. Finally, we consider combinations of only speckle features $[P_1, P_2]$, $[P_1, \sigma _{\textrm{P1}},P_2, \sigma _{\textrm{P2}}]$. To compare the feasibility of these combinations to distinguish brain glioma and intact tissue and study the applicability of speckle features, we apply LDA. It is worth noting that we choose LDA only as a suitable and convenient instrument for our studies, not aimed at finding the best approach and classification model for tissue distinguishing based on the suggested combination of features. We also admit that the sample set is limited and should be enlarged to perform accurate training and validation of models. However, we leave the problem of finding the best model beyond the scope of this study. Instead, we would like to investigate the advantages of speckle features and their combinations with attenuation features obtained for OCT images of brain tissues as the possible way for improving diagnostic potential of endoscopic OCT system.

Therefore, we briefly describe LDA approach used in this work. We consider ${\textbf{x}}$ to be the feature vector. In 1D, 2D, 3D or 4D space, we find a projection of data ${\textbf{x}}$ from two classes of points in 1D that maximizes the criterion

7 $$\begin{aligned} J({\textbf{w}})= & {} \frac{{\textbf{w}}^{\textsf{T}} S_{\textrm{B}} {\textbf{w}}}{{\textbf{w}}^{\textsf{T}} S_{\textrm{W}} {\textbf{w}}}, \end{aligned}$$

8 $$\begin{aligned} S_{\textrm{B}}= & {} ({\textbf{m}}_2 - {\textbf{m}}_1) ({\textbf{m}}_2 - {\textbf{m}}_1)^{\textsf{T}},\end{aligned}$$

9 $$\begin{aligned} S_{\textrm{W}}= & {} \sum _{n_1} ({\textbf{x}}_{\mathrm {n_1}} - {\textbf{m}}_1) ({\textbf{x}}_{\mathrm {n_1}} - {\textbf{m}}_1)^{\textsf{T}} + \sum _{n_2} ({\textbf{x}}_{\mathrm {n_2}} - {\textbf{m}}_2) ({\textbf{x}}_{\mathrm {n_2}} - {\textbf{m}}_2)^{\textsf{T}}, \end{aligned}$$

where ${\textbf{w}}^{\textsf{T}}$ is the projection operator, ${\textbf{m}}_i$ is the mean vector for class *i*, $S_{\textrm{B}}$ and $S_{\textrm{W}}$ are between- and within-class scatterers, respectively. Then we find a predicted border in the obtained 1D probability density space that separates two classes of points. Its position is conditioned with maximization of the sum of sensitivity and specificity $(\textrm{Se} + \textrm{Sp})$ assuming the actual diagnosis of each tissue sample. Sensitivity, specificity and precision metrics are calculated as

10 $$\begin{aligned} \textrm{Se}= & {} \frac{\textrm{TP}}{\textrm{TP} + \textrm{FN}}, \end{aligned}$$

11 $$\begin{aligned} \textrm{Sp}= & {} \frac{\textrm{TN}}{\textrm{TN} + \textrm{FP}}, \end{aligned}$$

12 $$\begin{aligned} \textrm{Pr}= & {} \frac{\textrm{TP}}{\textrm{TP} + \textrm{FP}}, \end{aligned}$$

where TP, TN, FP, and FN stand for true positive, true negative, false positive, and false negative errors of tissue discrimination, respectively. The *k*-fold cross validation of thus obtained LDA model is performed for each pair of tissue types; therefore, the metrics are finally averaged. We should note that during the sample splitting, the all images from a certain patient were used either for training or validation; thus, *k* depends on the number of patients in each pair of tissue types and is selected as much as possible.

The results of applying each feature combination for LDA are listed in Tables 2 and 3. For glioma grade 1 and 2 several combinations of features provide rather good performance. If one tries to find a single combination with high metrics in each case, it is possible to choose $[\mu , P_1, P_2]$. It is characterized by the metrics $>0.95$. Thus, a combination of attenuation coefficient with speckle features helps to increase to some extent the ability of solely attenuation properties to differentiate these tissues. Oppositely, for high grades, different combinations provide satisfactory results in each pair of tissues. Despite the metrics are lower for high grades comparing to low grades, it is clear that attenuation coefficient can be used only for white matter vs glioma grade 4 distinguishing, while in other cases $P_1$ and $[P_1, P_2]$ provide better results.

**Table 2:** The metrics of distinguishing between glioma of low grades and intact brain tissues.

Features	Cortex – glioma 1			Cortex – glioma 2			White matter – glioma 1			White matter – glioma 2		
	Se	Sp	Pr	Se	Sp	Pr	Se	Sp	Pr	Se	Sp	Pr
$\mu$	0.929	0.850	0.932	1.000	0.900	0.958	1.000	1.000	1.000	1.000	1.000	1.000
$[\mu , \sigma _{\mu }]$	0.928	0.864	0.928	0.989	0.900	0.958	1.000	1.000	1.000	1.000	1.000	1.000
$P_1$	1.000	1.000	1.000	0.796	0.946	0.968	0.875	0.812	0.906	0.693	0.516	0.619
$[P_1,\sigma _{\textrm{P1}}]$	0.938	1.000	1.000	0.818	0.871	0.934	0.844	0.906	0.941	0.975	0.915	0.947
$P_2$	0.938	1.000	1.000	0.954	0.946	0.968	0.938	1.000	1.000	0.954	1.000	1.000
$[P_2,\sigma _{\textrm{P2}}]$	0.920	1.000	1.000	0.904	0.982	0.989	0.969	0.500	0.826	0.940	0.482	0.763
$[\mu , P_1]$	0.875	1.000	1.000	1.000	0.900	0.958	1.000	0.969	0.983	1.000	1.000	1.000
$[\mu , P_2]$	0.848	0.900	0.962	0.989	0.925	0.968	1.000	1.000	1.000	1.000	1.000	1.000
$[P_1, P_2]$	1.000	1.000	1.000	0.954	1.000	1.000	1.000	0.875	0.928	0.954	0.953	0.968
$[\mu , \sigma _\mathrm {\mu },P_1]$	0.812	1.000	1.000	0.966	0.900	0.958	0.938	1.000	1.000	1.000	1.000	1.000
$[\mu , \sigma _\mathrm {\mu },P_2]$	1.000	0.929	0.956	1.000	0.900	0.958	1.000	1.000	1.000	1.000	1.000	1.000
$[\mu , P_1, P_2]$	**1.000**	**1.000**	**1.000**	**0.954**	**0.975**	**0.989**	**1.000**	**1.000**	**1.000**	**1.000**	**1.000**	**1.000**
$[\mu , \sigma _\mathrm {\mu },P_1,\sigma _{\textrm{P1}}]$	0.719	1.000	1.000	0.989	0.900	0.958	0.906	0.969	0.983	1.000	1.000	1.000
$[\mu , \sigma _\mathrm {\mu },P_2,\sigma _{\textrm{P2}}]$	1.000	0.964	0.972	1.000	0.900	0.958	1.000	1.000	1.000	1.000	1.000	1.000
$[P_1,\sigma _\mathrm {P_1},P_2,\sigma _{\textrm{P2}}]$	0.812	1.000	1.000	0.943	1.000	1.000	0.969	0.719	0.881	0.966	0.949	0.968

Significant values are in bold.

**Table 3:** The metrics of distinguishing between glioma of high grades and intact brain tissues.

Features	Cortex – glioma 3			Cortex – glioma 4			White matter – glioma 3			White matter – glioma 4		
	Se	Sp	Pr	Se	Sp	Pr	Se	Sp	Pr	Se	Sp	Pr
$\mu$	0.702	0.757	0.836	0.374	0.726	0.788	0.906	0.554	0.710	**0.886**	**0.950**	**0.978**
$[\mu , \sigma _{\mu }]$	0.764	0.622	0.781	0.469	0.469	0.734	0.854	0.683	0.804	0.876	0.938	0.973
$P_1$	0.913	0.900	0.944	**0.872**	**0.928**	**0.972**	0.945	0.714	0.833	0.666	0.500	0.840
$[P_1,\sigma _{\textrm{P1}}]$	0.757	0.928	0.938	0.880	0.700	0.905	0.882	0.714	0.833	0.763	0.164	0.697
$P_2$	0.754	0.150	0.544	0.285	0.731	0.716	0.622	0.317	0.490	0.132	0.857	0.758
$[P_2,\sigma _{\textrm{P2}}]$	0.507	0.479	0.566	0.238	0.649	0.633	0.833	0.536	0.783	0.749	0.500	0.869
$[\mu , P_1]$	0.882	0.700	0.566	0.869	0.914	0.967	0.945	0.750	0.848	0.876	0.631	0.856
$[\mu , P_2]$	0.802	0.679	0.820	0.345	0.606	0.746	0.875	0.518	0.692	0.936	0.866	0.948
$[P_1, P_2]$	**0.945**	**0.928**	**0.950**	0.852	0.928	0.972	**0.913**	**0.785**	**0.863**	0.611	0.498	0.805
$[\mu , \sigma _\mathrm {\mu },P_1]$	0.820	0.657	0.789	0.856	0.871	0.961	0.945	0.750	0.848	0.863	0.616	0.859
$[\mu , \sigma _\mathrm {\mu },P_2]$	0.826	0.614	0.771	0.528	0.360	0.734	0.792	0.692	0.762	0.926	0.866	0.947
$[\mu , P_1, P_2]$	0.945	0.736	0.848	0.843	0.808	0.932	0.945	0.750	0.848	0.939	0.732	0.903
$[\mu , \sigma _\mathrm {\mu },P_1,\sigma _{\textrm{P1}}]$	0.820	0.728	0.826	0.861	0.637	0.882	0.913	0.714	0.833	0.818	0.602	0.843
$[\mu , \sigma _\mathrm {\mu },P_2,\sigma _{\textrm{P2}}]$	0.743	0.614	0.763	0.534	0.343	0.718	0.858	0.572	0.787	0.920	0.488	0.863
$[P_1,\sigma _\mathrm {P_1},P_2,\sigma _{\textrm{P2}}]$	0.820	0.764	0.864	0.863	0.643	0.888	0.882	0.607	0.800	0.854	0.362	0.802

Significant values are in bold.

**Figure 7:**
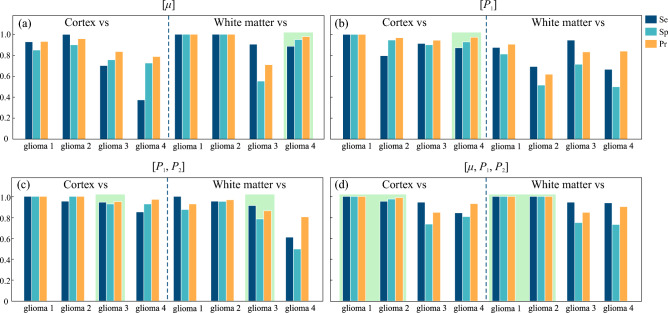
The metrics of distinguishing between glioma and intact brain tissues by means of (**a**) attenuation coefficient $\mu$; (**b**) speckle feature $P_1$; (**c**) combination $[P_1, P_2]$, (**d**) joint combination $[\mu , P_1, P_2]$. Green boxes indicate the cases, where the selected set of features are most appropriate.

**Figure 8:**
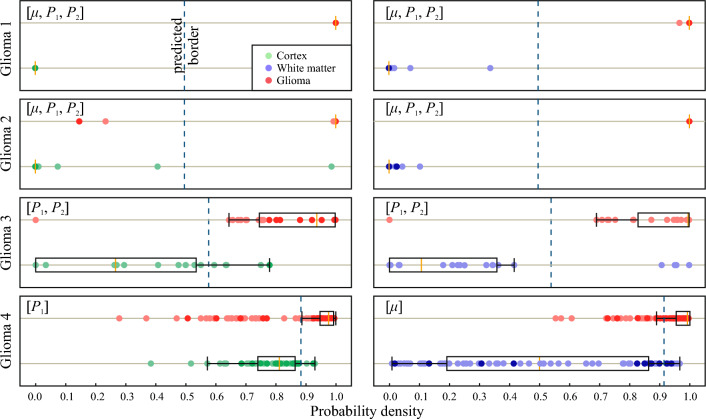
The distinguishing between glioma and intact tissues by means of LDA and the selected set of features. The dots are colored in accordance with the H&E-stained histology of the corresponding tissue samples. Orange lines show the medians of sample distributions, boxes show their 25th ($Q_1$) and 75th ($Q_3$) percentiles, whiskers denote $Q_1 - 1.5(Q_3 - Q_1)$ and $Q_3 + 1.5(Q_3 - Q_1)$.

**Figure 9:**
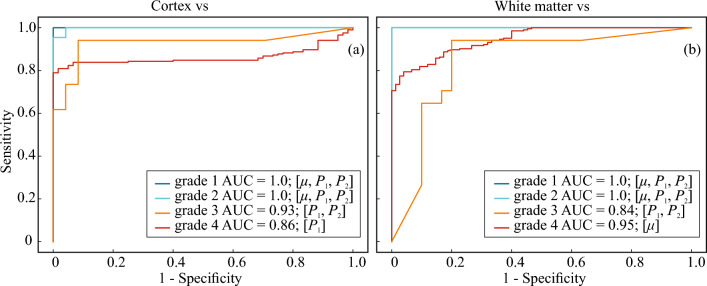
ROC curves analysed for LDA with the selected combination of features performed for distinguishing glioma tissues of all grades from (**a**) intact cortex and (**b**) white matter tissues.

The histograms of calculated metrics of tissue distinguishing by the selected combinations $\mu$, $P_1$, $[P_1, P_2]$ and $[\mu , P_1, P_2]$ are shown in Fig. 7. This figure clearly demonstrates several drawbacks of using attenuation coefficient for OCT-based glioma detection, as well as advantages of including speckle features in the analysis of OCT images. The results of performing LDA by the selected combinations of features in each pair of tissues are shown in Fig. 8. Here, the projections of actual dot clouds on the 1D probability density axis and the predicted discrimination border averaged during cross validation are demonstrated. Figure 8 illustrates that there is still a big challenge of distinguishing between glioma grade 4 and intact tissues. While $\mu$ remains the most appropriate feature among the considered combinations for the case white matter vs glioma grade 4, application of $P_1$ for cortex vs glioma grade 4 provides moderate overlap. This fact can be also discovered from Figs. 3d and 5d, where localization of dot clouds belonging to glioma grade 4 is better for $P_1$ than for $\mu$. Meanwhile, application of speckle features are reasonable in other cases. Figure 9 shows ROC curves of LDA model performed with the features selected for each pair of tissues. The area under curve (AUC) values also justify high accuracy of using joint attenuation-speckle feature space for distinguishing between intact tissues and glioma grade 1 and 2.

## Discussion

LDA of optical properties confirms the weakness of OCT to distinguish high-grade glioma tissue from intact cortex and white matter using attenuation-based approach. Sensitivity is extremely low for the case cortex vs glioma grade 4 (less than 0.5 for using both $\mu$ and $[\mu , \sigma _\mathrm {\mu }]$). The only case, where these features can be appropriate is white matter vs glioma grade 4. However, even for this pair of tissue types, sensitivity is less than 0.9. Combination of attenuation with its local dispersion helps to slightly improve some metrics in comparison with using $\mu$ in case of high grades; while for low grades, the performance is high for both $\mu$ and $[\mu , \sigma _\mathrm {\mu }]$.

In contrast, WA of OCT speckle properties allows us to enhance the performance of attenuation-based approach applied for brain glioma diagnosis. We have considered the first and second wavelet decomposition levels for acquisition of speckle properties and compare them between each other. When $P_1$ and $P_2$ are separately applied for tissue distinguishing, the results are more accurate for $P_2$ in case of low grades and $P_1$ – of high grades. Adding $\sigma _{\textrm{P1}}$ to $P_1$ is reasonable for moderate improvement of Se for cortex vs glioma grade 2 and 4, Sp for cortex vs glioma grade 3, Sp and Pr for white matter vs glioma grade 1 and significant enhancement of all metrics for white matter vs glioma grade 2. At the same time, adding $\sigma _{\textrm{P2}}$ to $P_2$ is beneficial for improvement of Sp and Pr for cortex vs glioma grade 2, Se and Pr for white matter vs glioma grade 3 and 4, Se for white matter vs glioma grade 1. However, combination $[P_1, P_2]$ can be also considered in several cases, namely cortex vs glioma grade 1, 2, and 3, while in other cases it reflects the contraversive performance of $P_1$ and $P_2$. Combination $[\mu , P_1, P_2]$ can be used for differentiation between low grades and intact tissues, since it improves the performance of attenuation features in case of intact cortex and maintain high sensitivity and specificity in case of white matter. Meanwhile, it is also possible to consider other combinations of attenuation and speckle features for low grades, but they don’t provide high accuracy for all tissue pairs.

To discriminate glioma tissue of high grades from intact cortex and white matter, application of speckle features is also reasonable, except for the case white matter vs glioma grade 4. However, the results are worse than obtained for low grades. The overall estimation of the sensitivity and specificity of the described attenuation, speckle and joint approaches shows that the diagnosis of high-grade glioma is still challenging for endoscopic OCT systems due to the infiltrative character and the presence of necrotic debris. To solve this problem it is possible to apply other features, but it may require additional instrumentation. For example, polarization-sensitive mode of OCT^23,56^ can be used for more careful detection of white matter, cortex and tumorous tissue artefacts. Depth-resolved variance of attenuation properties, which stems from the tissue heterogeneity, might be also considered for tissue differentiation. From the other hand, recent studies of glioma classification based on texture features demonstrated rather high performance^57^. This approach was applied for glioma grade 4 discrimination from intact brain tissues, but it might be useful to transfer it to grade 3. In this regard, recent development of machine learning algorithms^25,58,59^ may enhance the existing and emerging OCT-based approaches of brain tumor diagnosis.

In the context of instrumentation utilized in the reported study, the use of a time-domain OCT system with a rather weak focusing can be mentioned as an advantage. Indeed, the time-domain principle did not introduce the signal decay caused by the roll-off effect, the distorting influence of which should be taken into account for spectral-domain OCT setups^60,61^. Similarly, the fairly large length of the focus waist due to weak focusing also did not give appreciable distortions within the rather limited depth range used for quantification of attenuation.

In this paper, we illustrate the advantage of combining attenuation and speckle features for discrimination between intact and glioma tissues. For this purpose, a limited set of samples were considered. Obviously, an extended data base of OCT images of brain tissues should be involved for further studies and more careful analysis. At the same time, the large dataset should be applied to train other possible classification models.

Since the above-presented results demonstrate the feasibility of brain tissue differentiation by including speckle features in the analysis of OCT images, it is the scope of further study to extend the considered approaches for tissue mapping.

## Conclusions

This paper concerns a problem of improving the discrimination between brain malignant glioma and intact tissues by means of OCT. In particular, finding specific features from OCT images is one of the possible solutions. In this regard, we suggested extraction of speckle properties of images, solely or in the combination with commonly used attenuation properties. We have demonstrated the application of these features and their combinations for distinguishing between ex vivo human brain tissues – malignant glioma of WHO grades from 1 to 4 and intact white matter and cortex. These features included the attenuation coefficient, the power of local brightness fluctuations at the 1st and 2nd wavelet decomposition levels and standard deviations of these parameters. The feasibility of adding speckle information in the analysis was studied by performing LDA classification. The results of this study confirmed the drawbacks of using attenuation-based approach and demonstrated the possibility of increasing the OCT performance for neurosurgical purposes by additional analysis of speckle properties extracted from OCT images. We have shown that combination $[\mu , P_1, P_2]$ is reasonable for low-grade glioma, since it yields high values of Se, Sp, Pr for tissue distinguishing; while it is reasonable to apply $P_1$ and $[P_1, P_2]$ for glioma grade 4 distinguishing from cortex and glioma grade 3 – from cortex and white matter, respectively. Our work reveals the feasibility of applying such speckle information for neurosurgical diagnosis.

## Data Availability

Data underlying the results of this paper are not publicly available at this time, but may be obtained from the corresponding authors PVA (aleksandrovapolina98@gmail.com) and IND (in.dolganova@gmail.com) upon reasonable request.
